# Plasma metabolomics analyses highlight the multifaceted effects of noise exposure and the diagnostic power of dysregulated metabolites for noise-induced hearing loss in steel workers

**DOI:** 10.3389/fmolb.2022.907832

**Published:** 2022-08-19

**Authors:** Xiuzhi Zhang, Ningning Li, Yanan Cui, Hui Wu, Jie Jiao, Yue Yu, Guizhen Gu, Guoshun Chen, Huanling Zhang, Shanfa Yu

**Affiliations:** ^1^ Department of Pathology, Henan Medical College, Zhengzhou, Henan, China; ^2^ Department of Scientific Research and Foreign Affairs, Henan Medical College, Zhengzhou, Henan, China; ^3^ Department of Occupational and Environmental Health, College of Public Health, Zhengzhou University, Zhengzhou, Henan, China; ^4^ Henan Institute for Occupational Health, Zhengzhou, Henan, China; ^5^ National Institute for Occupational Health and Poison Control, Chinese Center for Disease Control and Prevention, Beijing, China; ^6^ Wugang Institute for Occupational Health, Wugang, Henan, China; ^7^ School of Public Health, Henan Medical College, Zhengzhou, Henan, China

**Keywords:** differential metabolomics, risk model, immune response, cell death, diagnosis, noise-induced hearing loss

## Abstract

Noise exposure can lead to various kinds of disorders. Noise-induced hearing loss (NIHL) is one of the leading disorders confusing the noise-exposed workers. It is essential to identify NIHL markers for its early diagnosis and new therapeutic targets for its treatment. In this study, a total of 90 plasma samples from 60 noise-exposed steel factory male workers (the noise group) with (NIHL group, *n* = 30) and without NIHL (non-NIHL group, *n* = 30) and 30 male controls without noise exposure (control group) were collected. Untargeted human plasma metabolomic profiles were determined with HPLC-MS/MS. The levels of the metabolites in the samples were normalized to total peak intensity, and the processed data were subjected to multivariate data analysis. The Wilcoxon test and orthogonal partial least square-discriminant analysis (OPLS-DA) were performed. With the threshold of *p* < 0.05 and the variable importance of projection (VIP) value >1, 469 differential plasma metabolites associated with noise exposure (DMs-NE) were identified, and their associated 58 KEGG pathways were indicated. In total, 33 differential metabolites associated with NIHL (DMs-NIHL) and their associated 12 KEGG pathways were identified. There were six common pathways associated with both noise exposure and NIHL. Through multiple comparisons, seven metabolites were shown to be dysregulated in the NIHL group compared with the other two groups. Through LASSO regression analysis, two risk models were constructed for NIHL status predication which could discriminate NIHL from non-NIHL workers with the area under the curve (AUC) values of 0.840 and 0.872, respectively, indicating their efficiency in NIHL diagnosis. To validate the results of the metabolomics, cochlear gene expression comparisons between susceptible and resistant mice in the GSE8342 dataset from Gene Expression Omnibus (GEO) were performed. The immune response and cell death-related processes were highlighted for their close relations with noise exposure, indicating their critical roles in noise-induced disorders. We concluded that there was a significant difference between the metabolite’s profiles between NIHL cases and non-NIHL individuals. Noise exposure could lead to dysregulations of a variety of biological pathways, especially immune response and cell death-related processes. Our results might provide new clues for noise exposure studies and NIHL diagnosis.

## Introduction

As a widespread occupational hazard, noise exposure leads to kinds of health concerns including hearing loss, hypertension, digestive disorders, cardiovascular diseases, and sleep disturbance in the exposed workers (Nari et al., 2020; Fredriksson et al., 2021; Jin et al., 2021). Among them, noise-induced hearing loss (NIHL) is one of the leading causes of occupational diseases and affects millions of people (Basner et al., 2014). In the past decades, a lot of researchers have focused their attention on the mechanisms of its occurrence. Plenty of gene variations including single-nucleotide polymorphisms (SNPs) of UBAC2 (rs3825427) (Wan et al., 2022), NRN1 (rs3805789), CAT (rs7943316) (Liu et al., 2021), CARD8 (Miao et al., 2021a), and CBX4 (rs1285250) (Wang et al., 2022) were reported to be associated with the risk of NIHL. In our previous studies, the associations of PON1, GSTP1, CDH23, and GPX1 with NIHL were also demonstrated (Li et al., 2020a; Li et al., 2020b; Wu et al., 2020). In a recent study, it was found that noise could promote cochlear hair cell apoptosis by inducing SP1/CBX4 pathway activation (Wang et al., 2022). As humans are unable to replace lost hair cells in the organ of Corti, the loss of hair cells would result in permanent deafness (Zheng and Zuo, 2017). In the process of industrial development, noise exposure cannot be completely avoided. Since the heterogeneity of individual susceptibility to noise exposure leads to different outcomes, early detection of susceptible people might avoid more serious injury to a certain extent. At present, NIHL diagnosis mainly depends on physical detection. Although it is feasible and operational for NIHL diagnosis, the early effects of noise exposure are unclear. The identification of new plasma biomarkers associated with NIHL and noise exposure would contribute to the improvement of the NIHL standard and provide new clues for a full understanding of its pathogenesis.

In a microarray analysis of microRNAs in male textile workers (Ding et al., 2016), the plasma levels of miR-16-5p, miR-24-3p, miR-185-5p, and miR-451a were uncovered to be upregulated in the NIHL patients than the exposed workers with normal hearing. However, compared with the controls without noise exposure, three of the microRNAs (miR-24, miR-185-5p, and miR-451a) were significantly downregulated in the workers with noise exposure. Based on these results, a positive association of the microRNAs with NIHL, while their negative association with noise exposure, could be deduced. The opposite associations of the microRNAs with NIHL and noise exposure indicated the complicated effects of noise exposure. Theoretically, there might be some biomarkers that would have consistent associations with NIHL and noise exposure or dysregulated solely in NIHL cases. So systemic studies were needed to identify reliable biomarkers for NIHL diagnosis and provide new clues for the noise exposure study.

As abnormalities in gene and protein levels would eventually be reflected in metabolism, metabolomics analysis was proposed (Huang et al., 2018). In fact, differential metabolomics analysis was found to be effective in the studies of various kinds of diseases and disorders including obesity, depressive disorder (Du et al., 2021), and cancerous disease (Brantley et al., 2022), indicating the variety of effects of noise exposure. In a recent study, plasma metabolomics analysis was performed on noise-exposed workers in a factory, and 20 metabolites were identified to be potential markers associated with NIHL (Miao et al., 2021b). However, as no controls without noise exposure was included, the associations of the metabolites with noise exposure could not be fully uncovered. Considering that there might be common effects of noise exposure on the exposed workers with and without NIHL, the inclusion of controls without noise exposure would provide more information about the metabolic changes associated with noise exposure. So far, there are few metabolomic studies on NIHL, and the reliability of the existing results needs to be verified in more studies. In addition, as different noises may also bring different impacts, the inclusion of more population in the study would be helpful for a better understanding of the impacts of noise exposure and provide new clues for NIHL detection and prevention.

In this present study, to identify the key metabolites and abnormal pathways associated with noise exposure and NIHL, untargeted metabolomics analyses of the plasma samples from occupational noise-exposed workers with and without NIHL in a steel factory and controls without noise exposure were performed. With the key metabolites, we constructed two risk models which could effectively discriminate the noise-exposed workers with and without NIHL. Furthermore, with cochlear gene expression profiles of mice with different susceptibilities to noise damage, the dysregulated genes associated with NIHL were investigated and their enrichment analyses were performed. Interestingly, there were some common pathways between enrichments of the dysregulated metabolites and the dysregulated genes. We hope these results would provide new clues for NIHL diagnosis and the study of noise exposure.

## Methods and materials

### Subjects, sample preparation, and metabolite identification

A total of 90 subjects including 60 noise-exposed workers (noise group) and 30 controls without noise exposure (control group) in a steel factory in China were selected into the study. According to their status of NIHL, the subjects in the noise group were divided into two subgroups: with NIHL (NIHL group, *n* = 30) and without NIHL (non-NIHL group, *n* = 30). Here, the sample size was calculated with the prevalence of NIHL in the factory (*p* = 0.20), the odds ratio of noise exposure in different groups (1.2), the type I error (*ɑ* = 0.05) and the type II error (*ß* = 0.20), and the case/control ratio (1:1). For the determination of the sample size, we also referred to the designs of similar studies (Banoei et al., 2019; Wang et al., 2020; Rothman et al., 2021). All workers were required to receive health examinations and hearing tests every 6 months. In order to collect important information about the individuals, we performed a questionnaire survey to obtain their family history, disease history, medication history, and some of their personal habits (smoking, drinking, and exercise). The inclusion criteria of noise-exposed workers were 1) male workers; 2) occupational noise exposure ≥85 dB(A); 3) with more than 3 years of accumulated occupational noise exposure; 4) for the NIHL group, with binaural average hearing threshold levels (HTLs) in high frequencies (3, 4, and 6 kHz) ≥ 40 dB; 5) for the non-NIHL group, the HTL of any one ear in linguistic frequencies (0.5, 1, and 2 kHz) < 25 dB and average binaural HTL in high frequencies <35 dB. The exclusion criteria of the noise-exposed workers were 1) patients with hearing loss or deafness caused by other diseases (Meniere’s disease, autoimmunological diseases, sudden deafness, etc.); 2) with exposure history to explosive noise; 3) with a family history of deafness; and 4) with diseases (otitis, tinnitus, craniocerebral injury, hypertension, diabetes, etc.) or ototoxic drug use (quinolones minoglycosides, vancomycin antibiotics, cisplatin, arsenic drugs, etc.) which could affect normal hearing. The controls were selected from the healthy administrative personnel who did not work in the front line and had no history of noise exposure and hearing loss in the same factory. The exclusion criteria of the controls were the same as those of the noise-exposed workers. The subjects in the NIHL group, non-NIHL group, and control group were matched with age, sex, smoking history, and drink history. For NIHL and non-NIHL workers, their jobs and seniority (difference <2 years) were also matched.

Informed consents were obtained in accordance with the Declaration of Helsinki. The work was reviewed and approved by the Ethics Committee of Henan Institute for Occupational Health (approval number: 2013003). The clinical features of the noise-exposed workers are shown in Supplementary Table S1.

**FIGURE 1:**
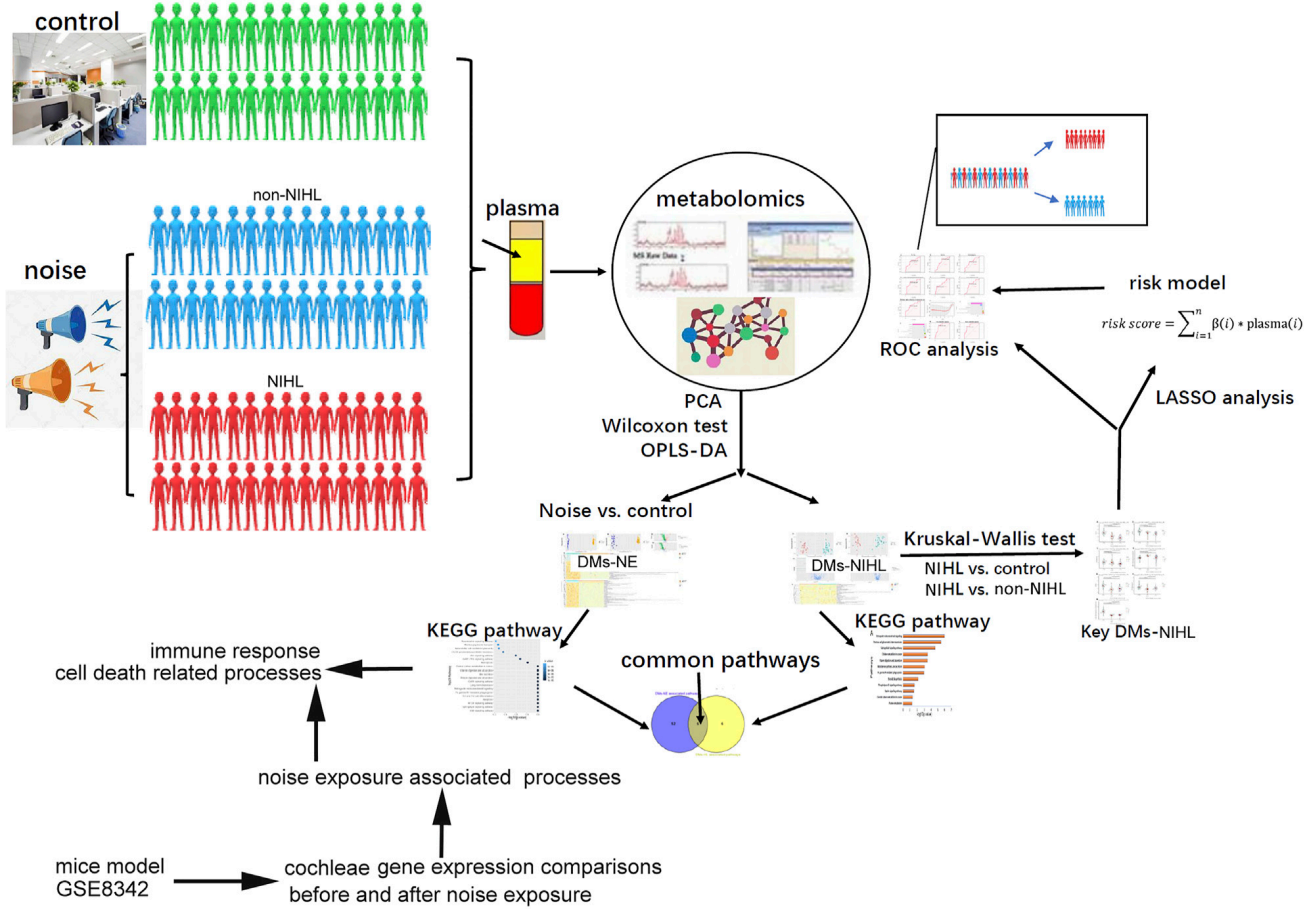
Overview of the study.

A hearing test was performed for the subjects, and their cumulative noise exposure (CNE) was estimated. The plasma samples from the subjects were prepared, and they were used for metabolite extraction and liquid chromatography-mass spectrometry (LC-MS) analysis to identify and quantify the metabolites in the samples. The specific procedures for this part are shown in Supplementary file 1 (method part), and the overview of the study is shown in Figure 1.

### Identification of differential metabolites between different groups

After normalization to total peak intensity, the processed data were log_2_(x) transformed and subjected to multivariate data analysis, including principal component analysis (PCA) and orthogonal partial least-squares discriminant analysis (OPLS-DA). The 10-fold cross-validation and response permutation testing (*n* = 200) was used to evaluate the robustness of the model (noise vs. control; NIHL vs. non-NIHL). The variable importance in the projection (VIP) value of each variable in the OPLS-DA model was calculated to indicate its contribution to the classification. For these analyses, R packages “FactoMineR” (https://cran.r-project.org/web/packages/FactoMineR/) and “ropls” (https://rdrr.io/bioc/ropls/) were used. In addition, metabolite comparisons were evaluated with an unpaired Wilcoxon test, and the metabolites with VIP >1 and *p* < 0.05 were considered significant. The differential metabolites between the noise group and control group were called differential metabolites associated with noise exposure (DMs-NE), while the ones between the NIHL group and the non-NIHL group were named differential metabolites associated with NIHL (DMs-NIHL). The profiles of the DMs-NE and DMs-NIHL were visualized with “pheatmap” (https://CRAN.R-project.org/package=pheatmap) package in R.

### Functional enrichment of the differential plasma metabolites associated with noise exposure and differential metabolites associated with NIHL

To investigate the pathways in which the DMs-NE and DMs-NIHL might be involved, functional enrichment analysis based on the Kyoto Encyclopedia of Genes and Genomes (KEGG) database was performed with the R package “KEGGREST” and “FELLA” package (method: diffusion) (Picart-Armada et al., 2018). The significant pathways and modules with *p* < 0.05 were extracted and visualized with the “ggplot2” package in R.

### Multiple comparisons of differential metabolites associated with NIHL and their diagnostic power in discriminating NIHL cases from non-NIHL cases

Considering the importance of early diagnosis of the NIHL patients, the DMs-NIHL were focused on for further analysis. To investigate the overall and pairwise differences of the metabolites among all the three groups, multiple comparisons were performed for the DMs-NIHL included in the KEGG enrichment analysis. The Kruskal–Wallis test was used with the “ggstatsplot” (https://CRAN.R-project.org/package=ggstatsplot) package in R, and Hommel’s method was used for the correction of the *p* values. The DMs-NIHL with significance in the comparisons of the NIHL group with the other two groups (NIHL vs. control; NIHL vs. non-NIHL) were included for receiver operating characteristics (ROC) analysis with the “pROC” package in R. Least absolute shrinkage and selection operator (LASSO) regression analysis was performed to construct risk models in noise-exposed individuals to estimate their possibility of suffering hearing loss. The risk model was set as follows:

$$risk\;score=\sum_{i=1}^{n}\text{β}\left(i\right)*\text{plasma}\left(i\right),$$

where n is the number of selected variables, *β* (*i*) is the coefficient of variable *i* in the multivariable Cox regression analysis, and plasma (*i*) is the plasma level of variable *i*. R packages “pROC” and “glmnet” were used for ROC and LASSO analyses.

### Further insight into the effects of noise exposure on the gene expression profiles of the cochleae of mice with different susceptibilities to noise damage

The gene expression profiles of the cochleae of 10 mice (GSE8342) from the GEO database (https://www.ncbi.nlm.nih.gov/geo/query/acc.cgi?acc=GSE8342) were used for analysis. In GSE8342 (Gratton et al., 2011), four mice (B6.CAST strain: control, *n* = 2; noise-exposed, *n* = 2) susceptible to NIHL and six mice resistant to NIHL (129X1/SvJ strain: control, *n* = 3; noise-exposed, *n* = 3) were included (Gratton et al., 2011). To explore the gene expression changes due to noise exposure, the cochlear gene expression comparisons of the two kinds of mice (B6.CAST strain and 129X1/SvJ strain, called B6 mice and 129X mice in this study, respectively) before and after noise exposure were estimated individually. The genes consistently dysregulated in the B6 mice and 129X mice were speculated to be common noise-induced gene expression changes, and enrichment analysis was performed to investigate their potential roles in NIHL development. Since the dysregulated genes unique for B6 mice or 129X mice might be associated with their differences in the hearing status after noise exposure, they were investigated to uncover their potential functions during noise exposure. The gene expression differences between the two kinds of mice before and after noise exposure were also investigated. The genes with significant differences between B6 mice and 129X mice after but not before noise exposure were extracted and analyzed for their potential function. The GEO2R tool (https://www.ncbi.nlm.nih.gov/geo/geo2r/) in the GEO database was used for gene expression comparisons. The genes with |log_2_ (fold change)| >1 (|logFC| >1) and *p* < 0.05 were considered significant. Enrichment analyses were performed to find the potential functions and their associated biological processes of the genes through Metascape (http://metascape.org/gp/index.html).

## Results

### Metabolite profiles of the samples

There were 7310 peaks and 5033 peaks detected in the positive and negative ion modes, respectively. A total of 1038 compounds (positive ion mode: 599, negative ion mode: 439) were identified to be known metabolites by matching the retention time, molecular weight, secondary fragmentation spectrum, and collision energy. The original data are given in Supplementary file 2.

Through PCA analysis, the metabolites’ profiles in the positive ion mode and the negative ion mode were investigated for their principal components (PCs) individually. With the first two PCs in the positive ion mode (Supplementary Figure S1A), the samples were separated into two major clusters: the “control” cluster and the “NIHL and non-NIHL” cluster. A similar result was also seen in the negative ion mode (Supplementary Figure S1B). It was indicated that compared with the samples in the control group, the samples in the noise group (NIHL group and non-NIHL group) presented significant different metabolite profiles, while the overall difference between the NIHL group and non-NIHL group was not so obvious both in the positive mode and the negative mode.

### Differential metabolites associated with noise exposure and their functional enrichments

**FIGURE 2:**
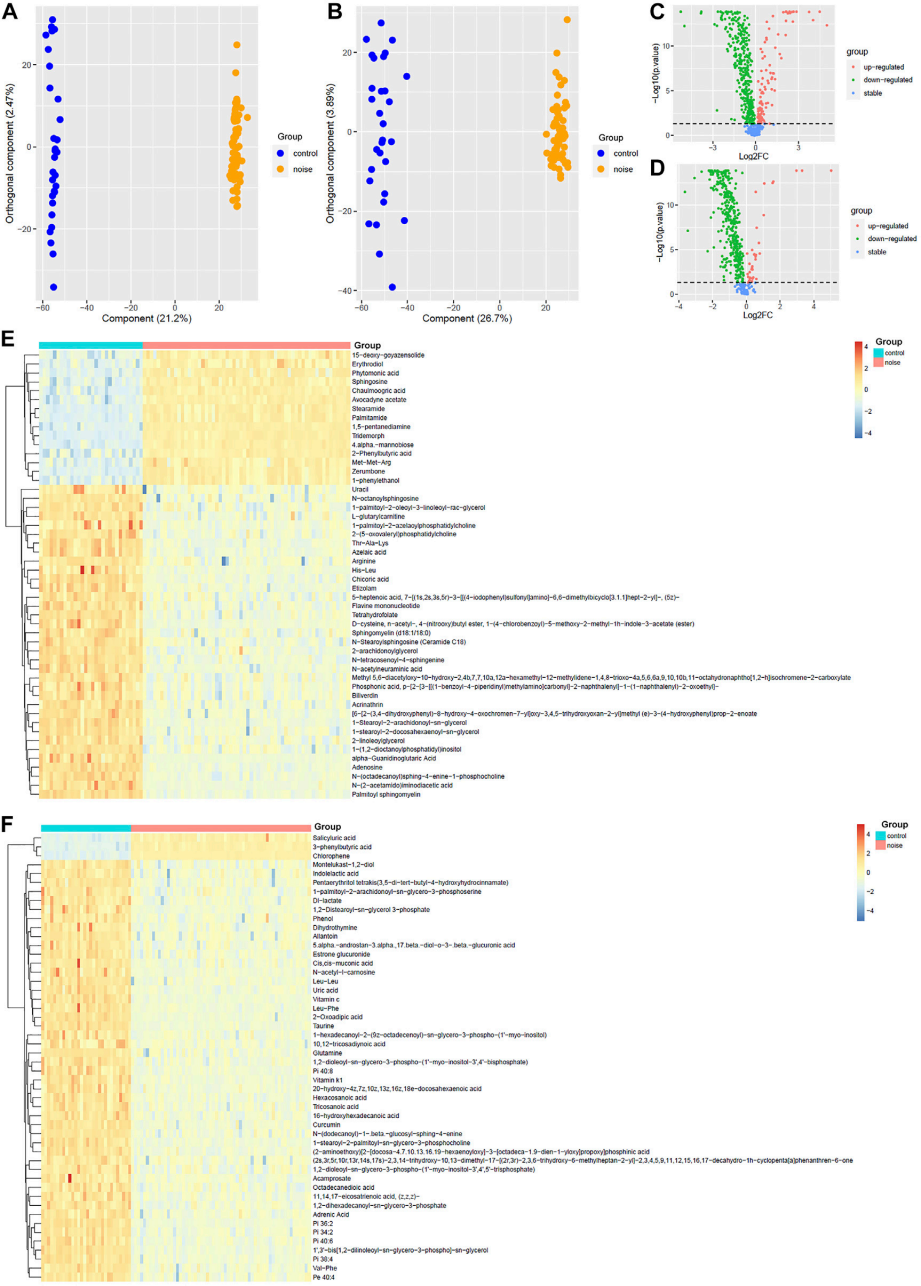
OPLS-DA of the plasma metabolites between the noise group and control group. **(A,B)** OPLS-DA score plots between the noise group and control group in positive and negative ion modes, respectively. **(C,D)** Comparisons of the metabolites between the noise group and the control group in the positive and negative mode, respectively. **(E,F)** Top 50 differential metabolites with the smallest *p* values and VIP >1 between the noise group and the control group in the positive ion mode and negative ion mode, respectively. The Wilcoxon test and OPLS-DA were used for analyses, and they were performed in R 4.0.3 software. The metabolites with *p* < 0.05 in the Wilcoxon test and VIP >1 in the OPLS-DA were considered significant. OPLS-DA, orthogonal partial least squares discrimination analysis; VIP, variable importance in projection; log_2_FC, log_2_(fold change).

As shown in Figure 2, OPLS-DA was effective in differentiating noise-exposed samples from the controls both in the positive mode and the negative mode (Figures 2A,B). With VIP >1 in the OPLS-DA model and *p* < 0.05 in the Wilcoxon test (Figures 2C,D), 469 DMs-NE including 53 upregulated and 416 downregulated metabolites were identified. The top 50 differential metabolites (with the smallest *p* values and VIP >1) in the positive mode and negative mode are shown in Figures 2E,F, respectively.

**FIGURE 3:**
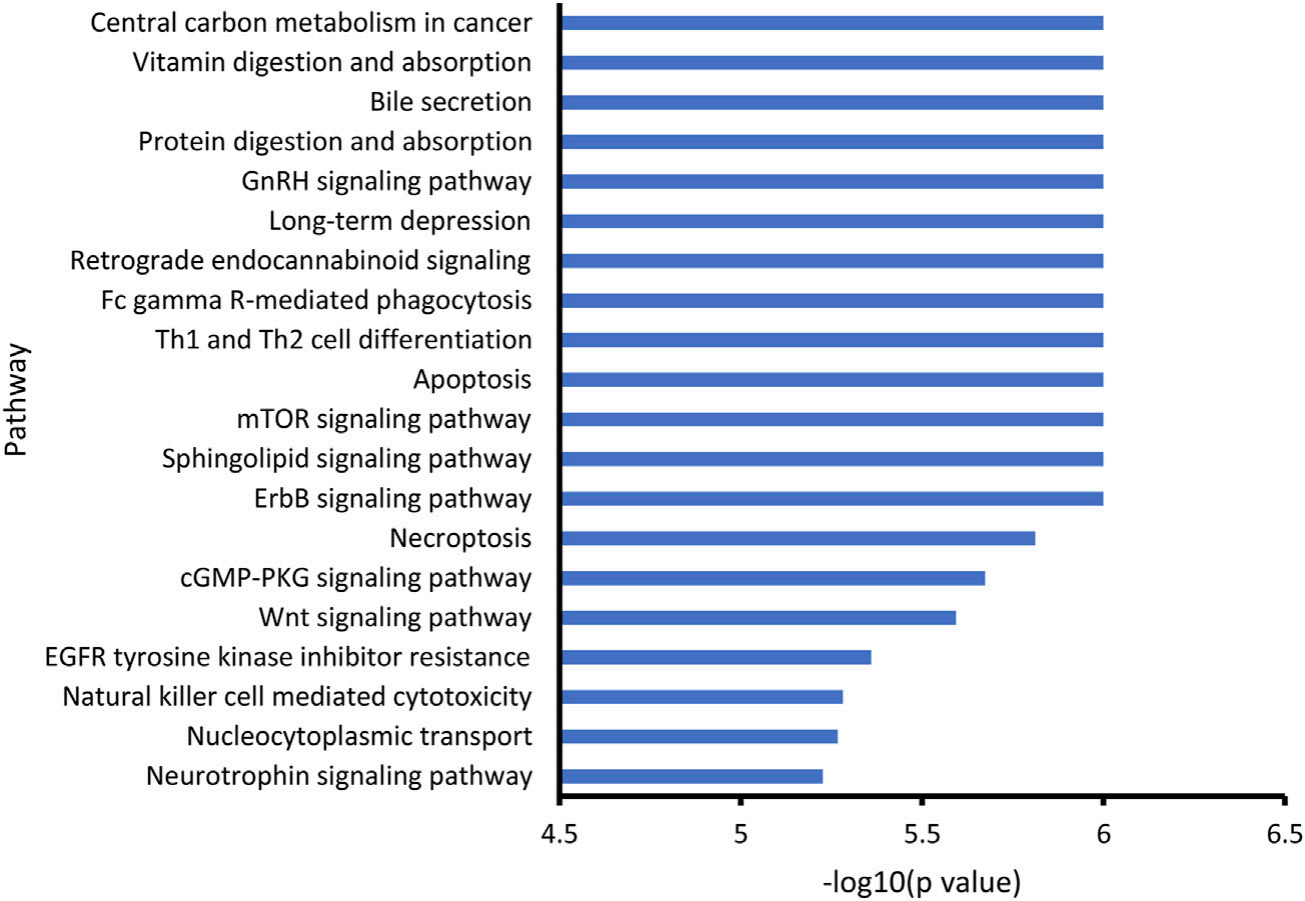
KEGG enrichment of noise-associated metabolites. R packages “KEGGREST” and “FELLA” were used for enrichment analysis. *p* < 0.05 was considered statistically significant.

Through KEGG enrichment analysis, the differential metabolites associated with noise exposure were significantly enriched in 58 KEGG pathways (Supplementary Table S2). The top 20 pathways with the smallest *p* values are shown in Figure 3. Among them, several signaling pathways, cell death-associated processes (apoptosis and necroptosis), immune response related processes (Th1 and Th2 cell differentiation, Fc gamma R-mediated phagocytosis, and natural killer cell-mediated cytotoxicity), long-term depression, and digestion and absorption were included, indicating the potential effects of noise exposure on a variety of biological processes.

### Differential metabolites associated with NIHL and their potential roles in the process of noise-induced hearing loss development

**FIGURE 4:**
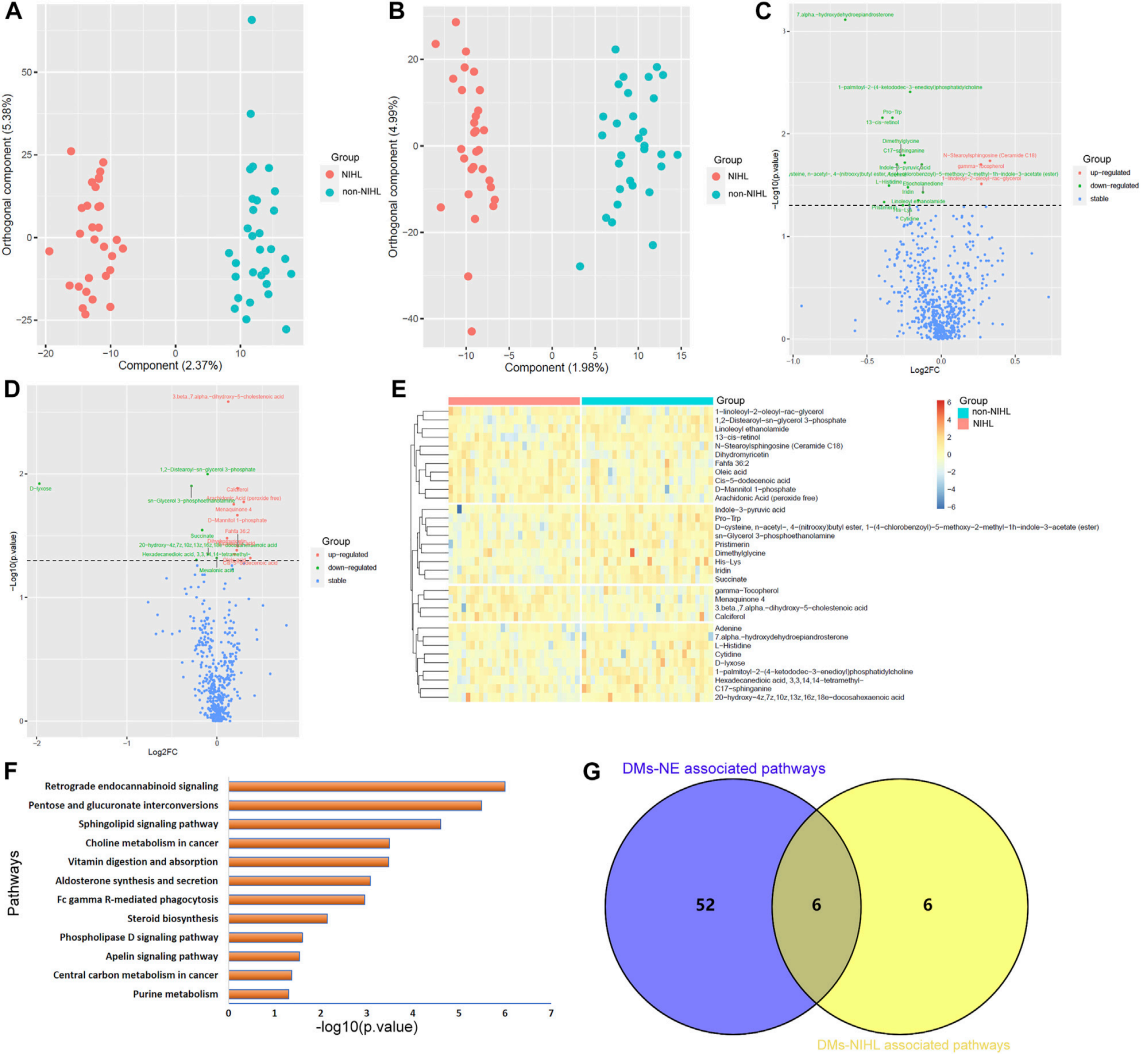
OPLS-DA of the plasma metabolites between the NIHL group and non-NIHL group. **(A,B)** OPLS-DA score plots between the NIHL group and non-NIHL group in positive and negative ion modes, respectively. **(C,D)** Comparisons of the metabolites between the NIHL group and the non-NIHL group in the positive and negative ion mode, respectively. **(E)** Expression profiles of the metabolites with *p* < 0.05 and VIP >1 (DMs-NIHL) in the NIHL samples and non-NIHL samples. **(F)** DMs-NIHL were significantly enriched in 12 KEGG pathways. **(G)** Six common pathways were associated with both DMs-NE and DMs-NIHL. The Wilcoxon test and OPLS-DA were used for analyses, and they were performed by R 4.0.3 software. The metabolites with *p* < 0.05 in the Wilcoxon test and VIP >1 in the OPLS-DA were considered significant. OPLS-DA, orthogonal partial least squares discrimination analysis; VIP, variable importance in projection; log_2_FC, log_2_(Fold change); NIHL, noise-induced hearing loss; non-NIHL, without NIHL. DMs-NE, differential metabolites associated with noise exposure; DMs-NIHL, differential metabolites associated with NIHL.

Unlike PCA, OPLS-DA identified the differences in the metabolite profiles of NIHL samples and non-NIHL samples and could separate the two groups clearly (Figures 4A,B). Through the Wilcoxon test, the metabolite comparisons between the NIHL group and non-NIHL group were evaluated (Figures 4C,D). With VIP >1 in the OPLS-DA model and *p* < 0.05 in the Wilcoxon test, 33 DMs-NIHL were identified. Among them, 11 metabolites were shown to be higher, while 22 metabolites were lower in the plasma of NIHL cases than those in the non-NIHL samples (Figure 4E)**.**

With the “FELLA” package in R, 17 of the 33 DMs-NIHL (Supplementary Table S3) were defined as compounds that belonged to the KEGG background and were included for enrichment analysis. As shown in Figure 4F, the 17 DMs-NIHL were significantly enriched in 12 pathways including retrograde endocannabinoid signaling, pentose and glucuronate interconversions, sphingolipid signaling pathway, choline metabolism in cancer, vitamin digestion and absorption, aldosterone synthesis and secretion, Fc gamma R-mediated phagocytosis, steroid biosynthesis, phospholipase D signaling pathway, apelin signaling pathway, central carbon metabolism in cancer, and purine metabolism. When intersected with the pathways associated with DMs-NE, six common pathways were shown (Figure 4G). The six pathways, including retrograde endocannabinoid signaling, sphingolipid signaling pathway, vitamin digestion and absorption, Fc gamma R-mediated phagocytosis, phospholipase D signaling pathway, and central carbon metabolism in cancer, might be associated with both noise exposure and NIHL. The dysregulations of these pathways might play crucial roles in the development of NIHL and other noise-associated disorders.

### Diagnostic power of the differential metabolites associated with NIHL in discriminating NIHL patients from non-NIHL cases

**FIGURE 5:**
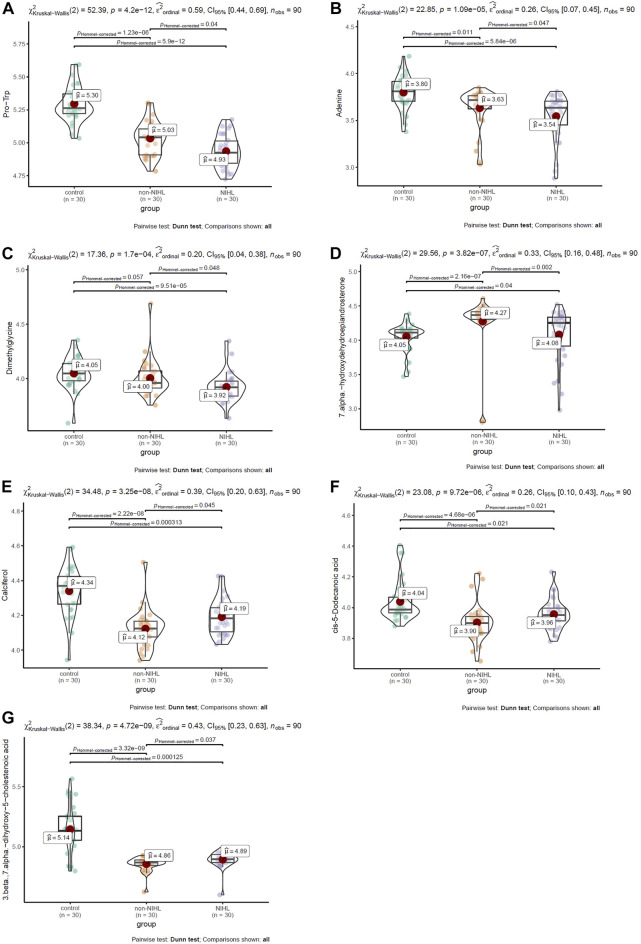
Multiple comparisons of DMs-NIHL between different groups. **(A–G)** Multiple comparisons of Pro-Trp, adenine, dimethylglycine, 7 alpha-hydroxydehydroepiandrosterone, calciferol, cis-5-dodecenoic acid, and 3 beta,7 alpha-dihydroxy-5-cholestenoic acid, respectively. DMs-NIHL, differential metabolites associated with noise-induced hearing loss. The Kruskal–Wallis test was used with the “ggstatsplot” package in R. Hommel-corrected *p* < 0.05 was considered statistically significant.

Multiple comparisons of the 17 DMs-NIHL mentioned previously were investigated for their differences among the NIHL group, non-NIHL group, and control group. With the threshold of Hommel-corrected *p* < 0.05, seven of the DMs-NIHL presented significant differences between the NIHL group and control group and non-NIHL group individually. As shown in Figures 5A–C, Pro-Trp, adenine, and dimethylglycine presented a gradual decrease trend among the control group, the non-NIHL group, and the NIHL group, and their lower levels were shown in the NIHL group than in the control group and the non-NIHL group. 7 Alpha-hydroxydehydroepiandrosterone was shown to be higher in the NIHL samples than in the control ones, while lower than the non-NIHL samples (Figure 5D). In contrast, calciferol, cis-5-dodecenoic acid, and 3 beta, 7 alpha-dihydroxy-5-cholestenoic acid were shown to be lower in the NIHL samples than those in the controls while higher than the non-NIHL samples (Figures 5E–G). Through ROC analysis, the diagnostic potentials of the seven DMs-NIHL were shown, with the area under the curves (AUCs) of 0.701, 0.674, 0.680, 0.748, 0.686, 0.649, and 0.723 for Pro-Trp, adenine, dimethylglycine, 7 alpha-hydroxydehydroepiandrosterone, calciferol, cis-5-dodecenoic acid, and 3 beta, 7 alpha-dihydroxy-5-cholestenoic acid, respectively (Supplementary Figure S2).

**FIGURE 6:**
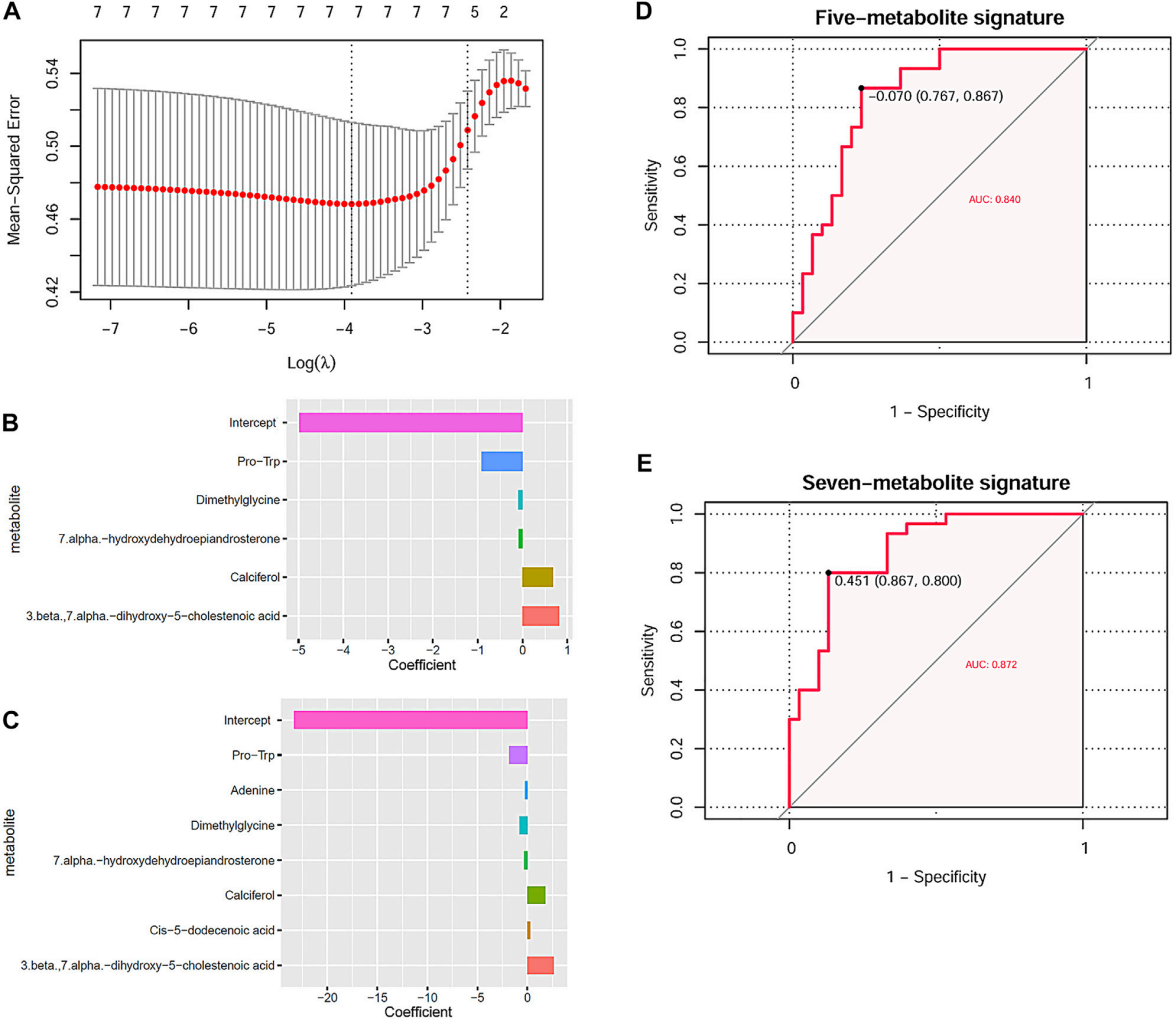
NIHL risk model construction through LASSO analysis and their diagnostic power. **(A)** Tuning parameter lambda (*λ*) selection using 10-fold cross-validation. **(B,C)** Five and seven DMs-NIHL with the absolute value of the coefficients >0 were included in the LASSO regression models. The *x*-axis represented the coefficients of the metabolites, and the *y*-axis indicated the metabolite variables. **(D,E)** LASSO regression models could predicate the hearing loss status effectively through ROC curve analysis. NIHL, noise-induced hearing loss; LASSO, least absolute shrinkage and selection operator; ROC, receiver operating characteristics; AUC, area under the curve.

Through LASSO regression analysis, two risk models of NIHL in noise-exposed individuals were constructed based on two appropriate lambda (λ) values (Figure 6A). The coefficients of the five-metabolite signature model and the seven-metabolite signature model are shown in Figures 6B,C, respectively. The two risk models are as follows:

*Risk score*_(five-metabolite)_ = −4.974 −0.913*Plasma_Pro-Trp_ −0.097*Plasma_Dimethylglycine_ −0.086*Plasma_7.alpha.-hydroxydehydroepiandrosterone_ + 0.678*Plasma_Calciferol_+ 0.815*Plasma_3.beta.,7.alpha.-dihydroxy-5-cholestenoic acid_

*Risk score*_(seven-metabolite)_ = −23.299–1.811*Plasma_Pro-Trp_ −0.232* Plasma_Adenine_ −0.803* Plasma_Dimethylglycine_ −0.335*Plasma_7.alpha.-hydroxydehydroepiandrosterone_ + 1.780*Plasma_Calciferol_ + 0.285*Plasma_Cis-5-dodecenoic acid_ + 2.634* Plasma_3.beta.,7.alpha.-dihydroxy-5-cholestenoic acid_

With the risk scores calculated with the risk models, the noise-exposed individuals could be differentiated effectively, with AUCs of 0.840 (Figure 6D) and 0.872 (Figure 6E) for the five-metabolite signature and the seven-metabolite signature, respectively. For the other 10 DMs-NIHL (Supplementary Figure S3), their significant difference was shown in either of the two comparisons (NIHL vs. control; NIHL vs. non-NIHL) only or neither of them.

### Gene dysregulations in mice cochleae due to noise exposure and their potential functions

**FIGURE 7:**
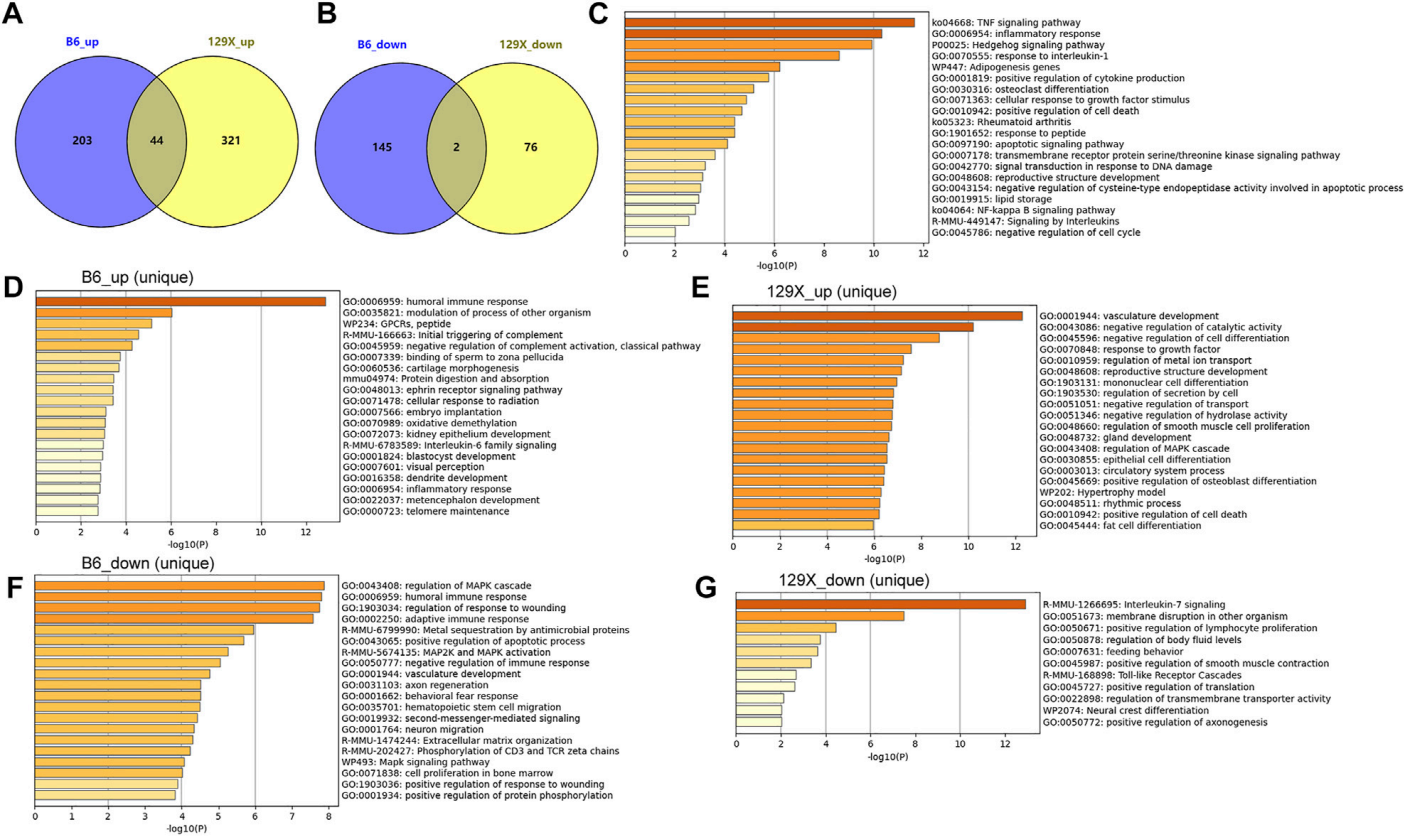
Dysregulated cochlear genes in mouse models due to noise exposure and their functional enrichment. **(A)** Gene numbers upregulated uniquely or consistently in the two kinds of mice due to noise exposure. **(B)** Gene numbers downregulated uniquely or consistently in the two kinds of mice due to noise exposure. **(C)** Pathways significantly associated with common upregulated genes in the susceptible B6 and resistant 129X mice due to noise exposure. **(D,E)** Pathways significantly associated with uniquely upregulated genes due to noise exposure in the susceptible B6 mice and resistant 129X mice, respectively. **(F,G)** Pathways significantly associated with uniquely downregulated genes due to noise exposure in the susceptible B6 mice and resistant 129X mice, respectively.

The cochlear gene expression changes of the mice before and after noise exposure were also investigated. With criteria of |logFC| >1 and *p* < 0.05, there were 394 genes upregulated (*n* = 247) or downregulated (*n* = 147) in the susceptible B6 mice due to noise exposure (Figure 7A). With regard to the resistant 129X mice, there were 365 genes upregulated and 78 genes downregulated after noise exposure (Figure 7B). There were 46 genes consistently higher (*n* = 44) or lower (*n* = 2, Il4 and Igbp1b) expressed in both the susceptible B6 mice and the resistant 129x mice (Figures 7A,B). With enrichment analysis, the 44 genes were shown to be associated with multiple biological processes (Figure 7C). Noticeably, several immune response-related pathways (TNF signaling pathway, inflammatory response, response to interleukin 1, positive regulation of cytokine production, and signaling by interleukins) and cell death-associated pathways (positive regulation of cell death, apoptotic signaling pathway, and negative regulation of the cysteine-type endopeptidase activity involved in the apoptotic process) were also included, consistent with the results of noise-associated metabolic changes in the human plasma in Figure 3, highlighting the potential effects of noise exposure on immune response and cell death.

In addition to the consistently dysregulated genes, there were more genes up- or downregulated only in either of the two kinds of mice (Figures 7A,B). There were 203 and 321 genes uniquely upregulated in B6 mice and 129X mice, respectively. There were 145 and 76 genes uniquely downregulated in B6 mice and 129X mice, respectively. The uniquely dysregulated genes were enriched in different biological processes (Figures 7D–G). In contrast to the cell death-related processes, interestingly, the 129X mice upregulated genes (Figure 7E) were uniquely enriched in some repair or anti-injury associated processes including vasculature development, negative regulation of catalytic activity, negative regulation of cell differentiation, and response to growth factor processes, which might account for their resistance to NIHL.

The differentially expressed genes between B6 mice and 129X mice before and after noise exposure were also investigated. In addition to the genes consistently expressed higher (*n* = 143) or lower (*n* = 173) in B6 mice than 129X mice, 241 expressional gene differences disappeared, while 143 and 173 genes emerged to be higher or lower expressed in B6 mice than in 129X mice after noise exposure (Supplementary Figures S4A,B). Interestingly, the genes emerged to be highly expressed in B6 mice than in 129X mice were enriched in positive regulation of the catabolic process and cell death response to oxidative stress processes, indicating the higher possibility of tissue injury and cell death in B6 mice than in 129X mice (Supplementary Figure S4C). These results also indicated the importance of the catabolic process and cell death in NIHL, consistent with the results in Figure 7D. In addition, the genes emerged to be expressed lower in B6 mice than those in 129X mice were enriched in positive regulation of the ATP metabolic process and negative regulation of neuron apoptotic processes (Supplementary Figure S4D), presenting the lower energetic production and higher cell death possibility in B6 mice than in 129 mice after noise exposure, also consistent with the aforementioned results.

## Discussion

NIHL has become one of the leading disorders confusing the noise-exposed workers. In a cross-sectional study, the prevalence of NIHL in the noise-exposed iron and steel workers was shown to be up to 48%, significantly higher than the controls (Nyarubeli et al., 2019). It is essential to explore the global dysregulations of metabolites in NIHL cases to identify new markers for its early diagnosis and new therapeutic targets for its treatment.

In previous studies, the overexposure of many physical and chemical pollutants (Fernandes et al., 2022; Gamrad-Streubel et al., 2022; Nie et al., 2022) was reported to be associated with the dysregulations of the immune system. In a recent study, the causal association between diesel exhaust exposure and dysfunction of immune response was confirmed, and the usefulness of metabolomic analysis was shown (Cheng et al., 2022). The multiple adverse impacts on the health of noise exposure were reported in plenty of studies (Essers et al., 2022; Wojciechowska et al., 2022), and its associations with immune response were also indicated. Consistently, in this study, a variety of biological processes associated with the DMs-NE were shown. The diversity of related pathways indicated the multifaceted influence of noise. It was reported that oxidative stress and inflammation were conducive to noise-induced disorders including NIHL (Tan et al., 2016; Daiber et al., 2020). Immune infiltrates were demonstrated to be associated with cochlear hair cell loss (Wood and Zuo, 2017). In a recent study, differential proteomics analysis has been used to find the key proteins associated with NIHL in mice cochleae, and several inflammation and autophagy-related proteins were identified (Miao et al., 2021c). In this study, through differential metabolomics analyses, the dysregulated plasma metabolites due to noise exposure were also found to be associated with immune response and cell death-related pathways, indicating their regulatory potential in the occurrence of noise-induced abnormalities. Since several signaling pathways, long-term depression, digestion, and absorption were also included in the enriched processes, we speculated that even for the noise-exposed workers without NIHL, there might be risks of other kinds of disorders.

Through differential plasma metabolomics analyses, Miao et al. (2021c) identified 20 NIHL-associated metabolites in noise-exposed workers in a factory which were significantly enriched in seven biological processes. Here, through differential plasma metabolomic analysis and enrichment analysis, 33 NIHL-associated metabolites were identified, and 12 biological processes with significance were shown. Surprisingly, few of the metabolites were consistent between the two studies. Interestingly, among the biological processes, the retrograde endocannabinoid pathway and choline metabolism were shown in both the studies, indicating their crucial roles during NIHL development in different populations. The differences between the results of the two studies might be explained by the population heterogeneity and different sample processing processes. Since no controls without noise exposure were included in the Miao study, the associations of the identified metabolites with noise exposure were unclear.

In the present study, with the controls without noise exposure as the background, the dysregulated metabolites and pathways due to noise exposure were identified. There were six common pathways that could be affected by both noise exposure and NIHL status. These pathways included retrograde endocannabinoid signaling, sphingolipid signaling pathway, vitamin digestion and absorption, Fc gamma R-mediated phagocytosis, phospholipase D signaling pathway, and central carbon metabolism in cancer. Endocannabinoids, also named as endogenous cannabinoids, are key modulators of synaptic function (Castillo et al., 2012). In addition to their neuromodulatory effects in the central nervous system, cannabinoid signaling was also reported to be critical for the development, maturation, function, and survival of cochlear hair cells (Ghosh et al., 2021). Sphingolipids, as essential constituents of the membrane in eukaryotic cells, were also crucial regulators of many cellular processes including apoptosis, proliferation, differentiation, autophagy, and migration inside the cells (Hannun and Obeid, 2018). The regulatory roles of sphingolipids in the hearing status were also shown in previous studies. It was reported that repression of local ceramide (one kind of sphingolipids) accumulation or suppression of the acid sphingomyelinase/ceramide pathway could ameliorate hearing loss and auditory cortex injury in noise-exposed mice (Su et al., 2019). In a study of noise-exposed workers in a candy manufacturing enterprise in China (Liang et al., 2021), the associations of digestive system disorders with noise exposure were also shown. The implications of neutrophil and macrophage infiltrations with noise-induced cochlear dysregulation were demonstrated in many studies (Mizushima et al., 2017; Hough et al., 2022). Considering the critical roles of the phospholipase D activity in efficient phagocytosis (Tanguy et al., 2019), here, the Fc gamma R-mediated phagocytosis and phospholipase D signaling pathway dysregulations might lead to the disorders of the two kinds of phagocytes in NIHL. As a hallmarker of cancerous diseases (Cífková et al., 2022), central carbon metabolism dysregulation was also shown in inner ear metabolism after noise exposure in mice models (Ji et al., 2019). Here, for the first time, we found the association of the central carbon metabolism dysregulation with NIHL through plasma metabolomics analyses, indicating its involvement in NIHL development.

Among the seven key metabolites associated with noise exposure and NIHL in this study, Pro-Trp, adenine, and dimethylglycine presented a gradual decrease trend among the control group, the non-NIHL group, and the NIHL group, with the lowest level in NIHL cases. Here, these dysregulations indicated their potential roles in noise-induced disorders. As the end products of the genome and proteome, many metabolites also have activities in the body. As a binary peptide, Pro-Trp could inhibit the function of dipeptidyl peptidase-IV (DPP-IV), a therapeutic target in type 2 diabetes (Nongonierma and FitzGerald, 2015). For adenine, its close relation to chronic kidney disease was reported in many studies (Cai et al., 2021; Kumakura et al., 2021). Interestingly, in contrast to its adverse effects on health, its function of inhibiting fatty liver development was also shown (Nishi et al., 2020). With regard to dimethylglycine, as a dietary supplement, dimethylglycine was reported to be able to enhance both humoral and cell-mediated immune responses in humans, indicating its immunoregulatory properties (Graber et al., 1981). In recent years, the effectiveness of dimethylglycine in improving the mental and physical status of children with autism spectrum disorder was also reported, indicating its implication in the regulation of the nervous system (Dhanjal et al., 2022). In addition, its antiulcer activity was shown in various rat models of ulcers (Hariganesh and Prathiba, 2000), indicating its anti-injury activity. In this study, we found the lower expression of dimethylglycine in NIHL cases than the non-NIHL workers and the normal controls. We speculated that noise exposure might affect the immune response, neural activity, and potentiality of the body cells against injury, through the dysregulation of dimethylglycine, which might be associated with NIHL and other noise-related disorders. Furthermore, the diagnostic efficiency of the dysregulated metabolites was evaluated. As a good method in machine learning, LASSO regression has been widely used in cancer marker studies (Hua et al., 2022; Koplewitz et al., 2022). In this study, with LASSO regression analysis, we constructed two risk models with the seven key metabolites for NIHL status predication. It was obvious that compared with individual metabolites, the metabolite signatures could provide higher efficiency, indicating their diagnostic values in NIHL detection in noise-exposed workers.

The gene expression dysregulations due to noise exposure and associated with NIHL were also investigated in mouse models. Through cochlear gene expression comparisons before and after noise exposure in mice models, we found that immune response-related pathways and cell death-related pathways were all affected by noise exposure both in the susceptible and resistant mice, consistent with the metabolic changes in the human plasma. This consistency enhanced the critical roles of the dysregulations of immune response and cell death in noise-induced disorders. In addition to the common gene expression changes, there were some genes uniquely dysregulated in either of the two kinds of mice. Compared with the susceptible mice, the genes uniquely increased in resistance mice after noise exposure was associated with vasculature development and response to growth factor processes, which were important for wound healing and tissue repair (Ferrigno et al., 2020; Groblewska and Mroczko, 2021). The consistent results were also shown in uniquely differentially expressed cochlear genes between the two kinds of mice after noise exposure. Compared with the resistant mice, the lower vulnerable characteristics and weak repair ability of susceptible mice might account for their susceptibility to NIHL.

Although there were plenty of studies of NIHL, most of them focused on the identification of susceptibility-related genes and SNPs. The pathogenic mechanisms of NIHL were not fully illustrated. It is needed to investigate the effects of noise exposure at global levels. As a kind of body fluids, plasma could reflect the metabolic dysregulations in the cells, tissues, and organs. In this study, the usefulness and reliability of plasma metabolomics analysis are shown. Through differential plasma metabolomics analyses, we identified 469 plasma DMs-NE and 58 KEGG pathways associated with noise exposure, indicating the complexity of its impacts. Among the pathways, immune response and cell death-related processes might be critical regulators in the development of noise-induced disorders. The 12 pathways associated with 33 DMs-NIHL might be implicated in the NIHL occurrence. The six common pathways associated with noise and NIHL would provide a new direction for the NIHL study. The seven key DMs-NIHL and the two risk models would be new markers for NIHL diagnosis. As some of the metabolites also have activities, further study is needed to explore their specific roles in NIHL development. Considering the associations of lower vulnerable characteristics and weak repair ability with the susceptibility to NIHL in mouse models, improvement of the anti-injury and repair ability might be useful for the prevention of the disorder.

## Data Availability

The original contributions presented in the study are included in the article/Supplementary Material; further inquiries can be directed to the corresponding author.
