# Supplementary material for: Plasma metabolomics analyses highlight the multifaceted effects of noise exposure and the diagnostic power of dysregulated metabolites for noise-induced hearing loss in steel workers
Source: Front Mol Biosci. 2022 Aug 19;9:907832. doi: 10.3389/fmolb.2022.907832 (PMC9437629; doi:10.3389/fmolb.2022.907832)
Supplement: Supplementary file 1 [file DataSheet1.PDF]

**Plasma metabolomics analyses highlight the multifaceted effects of noise-exposure and the diagnostic power of dysregulated metabolites for noise-induced hearing loss in steel workers**

**Supplementary file 1 (Methods part)**

**Hearing test and cumulative noise exposure (CNE) estimation**

After more than 12 hours away from the occupational noise environment, the subjects were required to take a binaural air and bone conduction threshold audiometry test (at 0.5, 1, 2, 3, 4, 6 kHz) performed by a professional doctor using 216 audiometers (Interacoustics AS Company, Denmark). The surrounding should be quiet, with the noise background < 25 dB(A). According to GB/T7582-2004, the original data of the tests were adjusted by age and sex. The noise exposure level was represented by CNE and it was calculated according to previous studies (Jiang et al., 2021; Xie et al., 2016).

**Plasma collection and metabolite extraction**

We collected 3ml morning fasting peripheral venous blood from each subject to a vacuum anticoagulant (heparin sodium) tube. Let it stand at room temperature for 30 minutes. After centrifuging at 2000 g for 10 minutes, the supernatant was separated and placed at -80°C until further determination. To extract metabolites from plasma samples, 400μL of the cold extraction solvent methanol/acetonitrile/H2O (2:2:1, v/v/v) was added to 100mg of each sample. After adequate vortexing, the samples were incubated on ice for 20 minutes, and then centrifuged at 14,000 g for 20 minutes at 4°C. The supernatant was collected and dried in a vacuum centrifuge at 4°C. For liquid chromatography-mass spectrometry (LC-MS) analysis, the samples were re-dissolved in 100μL acetonitrile/water (1:1, v/v) solvent and transferred to LC vials.

**LC-MS Analysis and data processing**

For untargeted metabolomics analysis of polar metabolites in the samples, the extracts were analyzed using a quadrupole time-of-flight mass spectrometer (Sciex TripleTOF 6600) coupled to hydrophilic interaction chromatography via electrospray ionization in Shanghai Applied Protein Technology Co., Ltd. LC separation was performed on a ACQUIY UPLC BEH Amide column (2.1mm × 100mm, 1.7μm particle size (waters, Ireland) using a gradient of solvent A (25 mM ammonium acetate and 25mM ammonium hydroxide in water) solvent B (acetonitrile). The gradient was 85% B for 1min and was linearly reduced to 65% in 11min, and then was reduced to 40% in 0.1min and kept for 4 min, and then increased to 85% in 0.1min, with a 5min re-equilibration period employed. Flow rate was 0.4 mL/minute, column temperature was 25°C, auto sampler temperature was 5°C, and injection volume was 2μL. The mass spectrometer was operated in both negative and positive ionization modes. The ESI source conditions were set as follows: Ion Source Gas1 (Gas1) as 60, Ion Source Gas2 (Gas2) as 60, curtain gas (CUR) as 30, source temperature: 600°C, IonSpray Voltage Floating (ISVF) ± 5500 V. In MS acquisition, the instrument was set to acquire over the m/z range 60-1000 Da, and the accumulation time for TOF MS scan was set at 0.20 s/spectra. In auto MS/MS acquisition, the instrument was set to acquire over the m/z range 25-1000 Da, and the accumulation time for product ion scan was set at 0.05 s/spectra. The product

ion scan is acquired using information dependent acquisition (IDA) with high sensitivity mode selected. The parameters were set as follows: the collision energy (CE) was fixed at 35V with ± 15eV; declustering potential (DP), 60V (+) and −60V (−); exclude isotopes within 4 Da, and candidate ions to monitor per cycle: 10.

The raw MS data (wiff.scan files) were converted to MzXML files using ProteoWizard MSConvert before importing into freely available XCMS software. For peak picking, the following parameters were used: centWave m/z = 25 ppm, peakwidth = c (10, 60), prefilter = c (10, 100). For peak grouping, bw = 5, mzwid = 0.025, minfrac = 0.5 were used. In the extracted ion features, only the variables having more than 50% of the nonzero measurement values in at least 4 one group were kept. Compound identification of metabolites by MS/MS spectra with an in-house database established with available authentic standards.

## Supplementary Tables and Figures

**Table S1**. The characteristics of the noise-exposed samples.

| | NIHL (n=30) | Non-NIHL(n=30) | *P* |
|---|---|---|---|
| CNE | 97.26 +/- 4.60 | 97.24 +/ 4.65 | 0.986 |
| Age(y) (Mean +/-SD) | 39.28 +/- 7.32 | 38.96 +/- 7.32 | 0.866 |
| Smoking (yes/no) | 16/14 | 16/14 | 1 |
| Drinking (yes/no) | 19/11 | 17/13 | 0.792 |
| Exercise (yes/no) | 10/20 | 13/17 | 0.595 |

Wilcoxon test and Char-square test was used for the comparisons of continuous data and qualitative data, respectively. $P$<0.05 was considered significant. Exercise (yes) indicates at least 60 minutes of aerobic exercise per week. NIHL, noise-induced hearing loss; non-NIHL, without NIHL; CNE, cumulative noise exposure; N, the number of the samples; SD, standard deviation.

**Table S2**. The 58 KEGG pathways associated with noise-exposure.

| KEGG.id | KEGG.name | p |
|---|---|---|
| hsa04012 | ErbB signaling pathway | 1.000E-06 |
| hsa04071 | Sphingolipid signaling pathway | 1.000E-06 |
| hsa04150 | mTOR signaling pathway | 1.000E-06 |
| hsa04210 | Apoptosis | 1.000E-06 |
| hsa04658 | Th1 and Th2 cell differentiation | 1.000E-06 |
| hsa04666 | Fc gamma R-mediated phagocytosis | 1.000E-06 |
| hsa04723 | Retrograde endocannabinoid signaling | 1.000E-06 |
| hsa04730 | Long-term depression | 1.000E-06 |
| hsa04912 | GnRH signaling pathway | 1.000E-06 |
| hsa04974 | Protein digestion and absorption | 1.000E-06 |
| hsa04976 | Bile secretion | 1.000E-06 |
| hsa04977 | Vitamin digestion and absorption | 1.000E-06 |
| hsa05230 | Central carbon metabolism in cancer | 1.000E-06 |

| | | |
|---|---|---|
| hsa04217 | Necroptosis | 1.542E-06 |
| hsa04022 | cGMP-PKG signaling pathway | 2.118E-06 |
| hsa04310 | Wnt signaling pathway | 2.553E-06 |
| hsa01521 | EGFR tyrosine kinase inhibitor resistance | 4.372E-06 |
| hsa04650 | Natural killer cell mediated cytotoxicity | 5.229E-06 |
| hsa03013 | Nucleocytoplasmic transport | 5.405E-06 |
| hsa04722 | Neurotrophin signaling pathway | 5.945E-06 |
| hsa04724 | Glutamatergic synapse | 5.979E-06 |
| hsa04660 | T cell receptor signaling pathway | 7.272E-06 |
| hsa04978 | Mineral absorption | 8.489E-06 |
| hsa05235 | PD-L1 expression and PD-1 checkpoint pathway in cancer | 1.002E-05 |
| hsa04024 | cAMP signaling pathway | 1.425E-05 |
| hsa04926 | Relaxin signaling pathway | 1.610E-05 |
| hsa04614 | Renin-angiotensin system | 2.962E-05 |
| hsa04935 | Growth hormone synthesis, secretion and action | 4.621E-05 |
| hsa04064 | NF-kappa B signaling pathway | 6.701E-05 |
| hsa04662 | B cell receptor signaling pathway | 8.488E-05 |
| hsa05170 | Human immunodeficiency virus 1 infection | 1.052E-04 |
| hsa04933 | AGE-RAGE signaling pathway in diabetic complications | 1.903E-04 |
| hsa05214 | Glioma | 2.929E-04 |
| hsa00232 | Caffeine metabolism | 3.178E-04 |
| hsa04015 | Rap1 signaling pathway | 3.283E-04 |
| hsa04621 | NOD-like receptor signaling pathway | 3.645E-04 |
| hsa04072 | Phospholipase D signaling pathway | 3.743E-04 |
| hsa04530 | Tight junction | 3.757E-04 |
| hsa05017 | Spinocerebellar ataxia | 3.802E-04 |
| hsa04972 | Pancreatic secretion | 6.158E-04 |
| hsa05131 | Shigellosis | 9.246E-04 |
| hsa04725 | Cholinergic synapse | 1.197E-03 |
| hsa05012 | Parkinson disease | 1.526E-03 |
| hsa04979 | Cholesterol metabolism | 1.725E-03 |
| hsa05340 | Primary immunodeficiency | 1.779E-03 |
| hsa05032 | Morphine addiction | 2.319E-03 |
| hsa04137 | Mitophagy - animal | 2.891E-03 |
| hsa04392 | Hippo signaling pathway - multiple species | 3.554E-03 |
| hsa04120 | Ubiquitin mediated proteolysis | 3.573E-03 |
| hsa05100 | Bacterial invasion of epithelial cells | 3.844E-03 |
| hsa04061 | Viral protein interaction with cytokine and cytokine receptor | 4.400E-03 |
| hsa04915 | Estrogen signaling pathway | 4.683E-03 |
| hsa03040 | Spliceosome | 5.270E-03 |
| hsa05034 | Alcoholism | 5.877E-03 |
| hsa00600 | Sphingolipid metabolism | 5.878E-03 |
| hsa05167 | Kaposi sarcoma-associated herpesvirus infection | 5.944E-03 |

| | | |
|---|---|---|
| hsa04620 | Toll-like receptor signaling pathway | 5.972E-03 |
| hsa05110 | Vibrio cholerae infection | 6.873E-03 |

**Table S3.** The 17 metabolites in the 33 DMs-NIHL defined as compounds belonged to the KEGG background

| KEGG | Name |
|---|---|
| C18045 | 7.alpha.-hydroxydehydroepiandrosterone |
| C00147 | Adenine |
| C01026 | Dimethylglycine |
| C00836 | Pro-Trp |
| C17335 | 3.beta.,7.alpha.-dihydroxy-5-cholestenoic acid |
| C05441 | Calciferol |
| C00476 | D-lyxose |
| C00387 | His-Lys |
| C00712 | Oleic acid |
| C00475 | Cytidine |
| C00828 | Menaquinone 4 |
| C00219 | Arachidonic Acid (peroxide free) |
| C00135 | L-Histidine |
| C00331 | Indole-3-pyruvic acid |
| C00644 | D-Mannitol 1-phosphate |
| C01233 | sn-Glycerol 3-phosphoethanolamine |
| C00042 | Succinate |

DMs-NIHL, differential metabolites associated with noise-induced hearing loss.

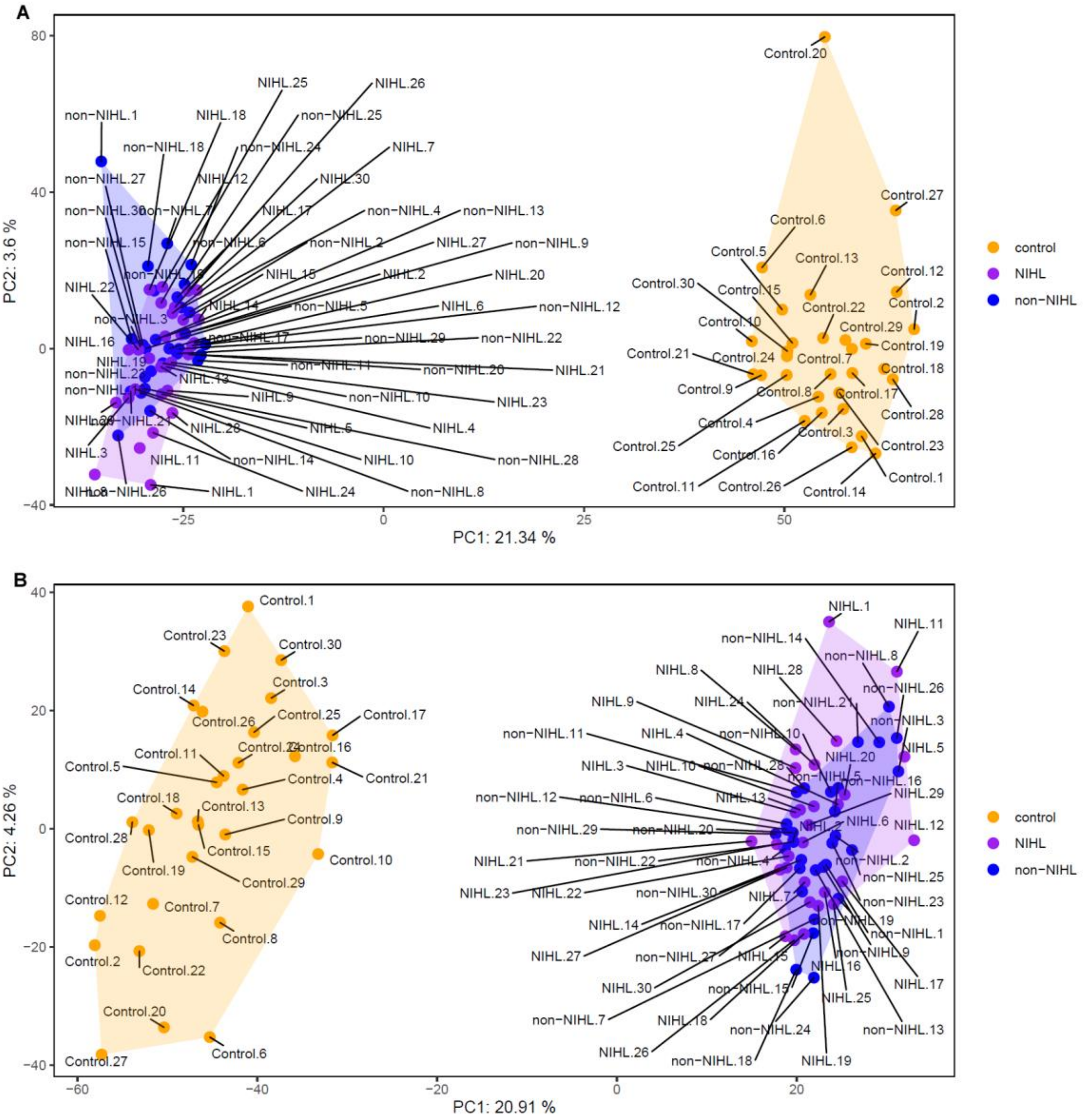


**Figure S1.** PCA of the metabolite profiles in the samples of different groups. (A) PCA of the metabolites in the positive ion group. (B) PCA of the metabolites in the negative ion group. PCA, principal components analysis. NIHL, noise-induced hearing loss; non-NIHL, without NIHL.

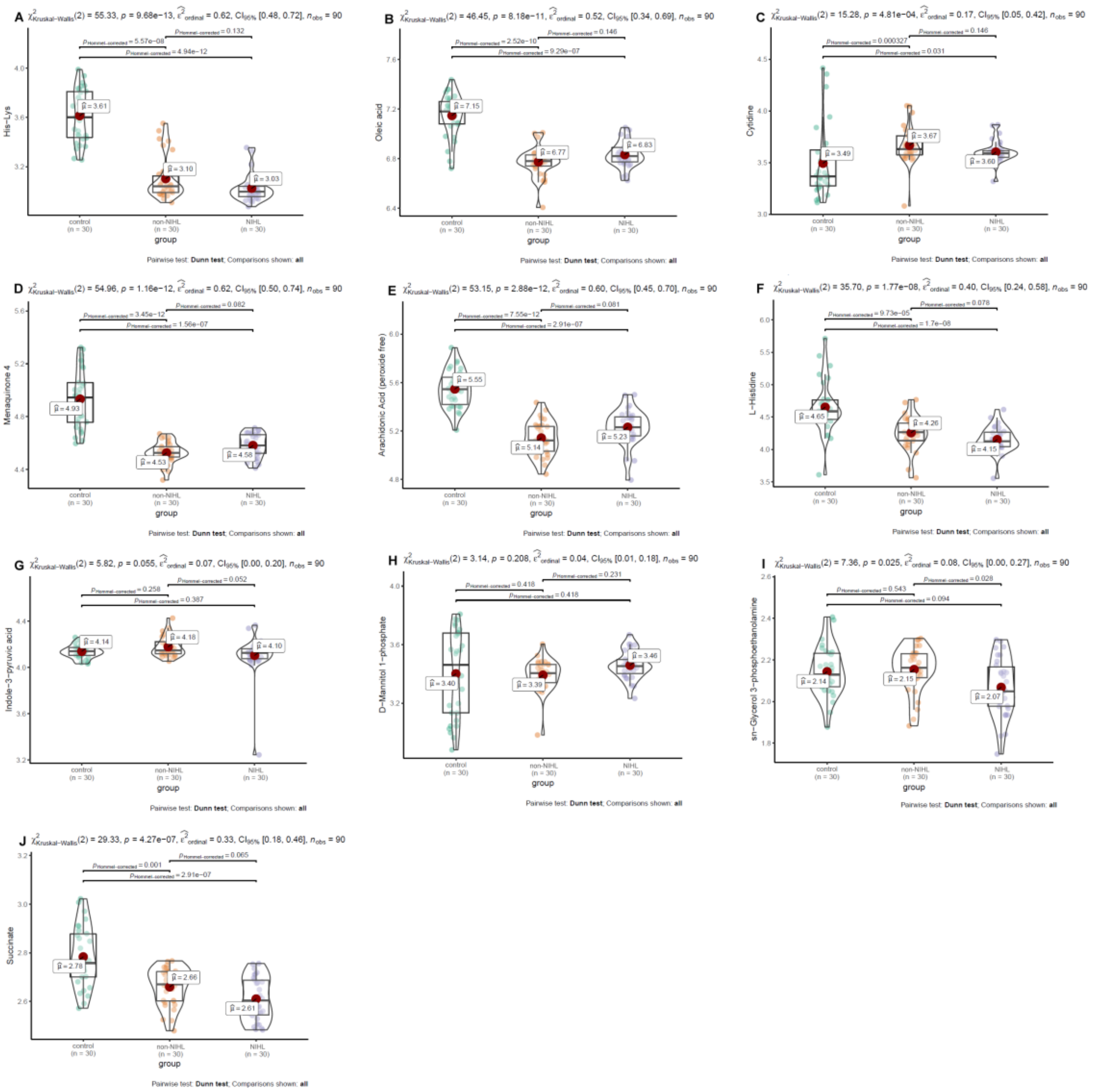


**Figure S2.** Multiple comparisons of DMs-NIHL between different groups. (A-J) Multiple comparisons of His-Lys, Oleic acid, Cytidine, Menaquinone 4, Arachidonic Acid (peroxide free), L-Histidine, Indole-3-pyruvic acid, D-Mannitol 1-phosphate, sn-Glycerol 3-phosphoethanolamine, and Succinate, respectively. DMs-NIHL, differential metabolites associated with noise-induced hearing loss. Kruskal-Wallis test was used with "ggstatsplot" package in R. Hommel-corrected $p < 0.05$ was considered significant.

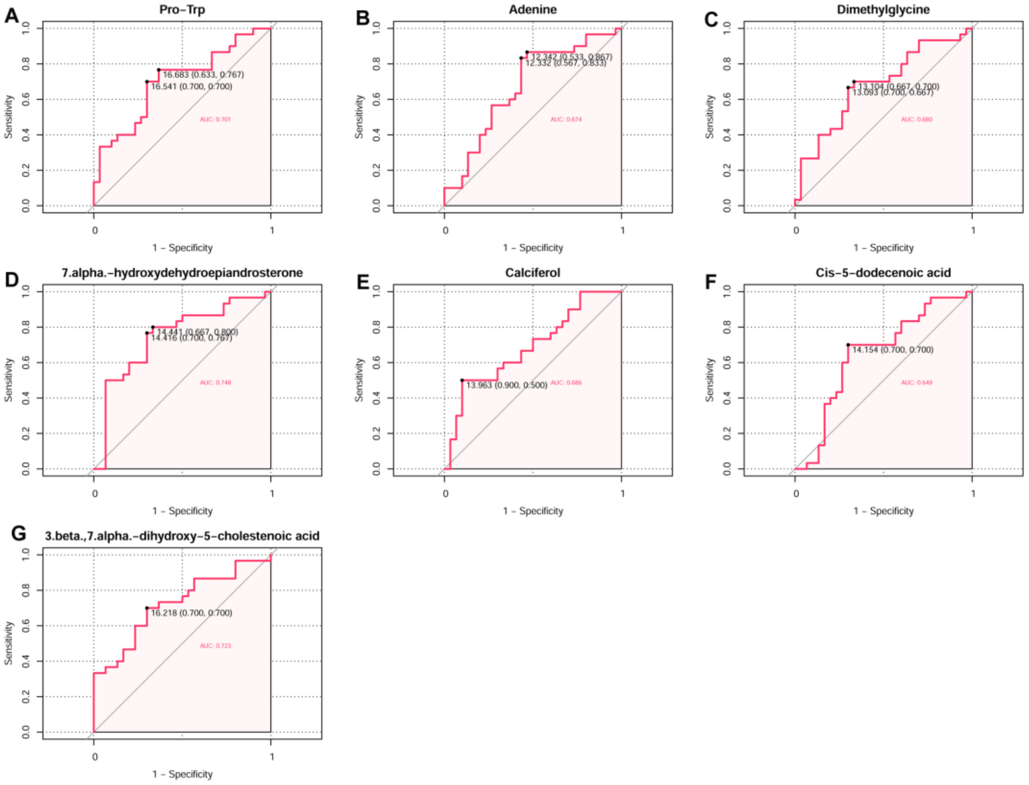


**Figure S3.** The ROC analysis of DMs-NIHL in discriminating NIHL group and non-NIHL group. (A-G) The ROC analysis of Pro-Trp, adenine, dimethylglycine, 7 alpha-hydroxydehydroepiandrosterone, calciferol, cis-5-dodecenoic acid, and 3 beta, 7 alpha-dihydroxy-5-cholestenoic acid, respectively. DMs-NIHL, differential metabolites associated with hearing loss. NIHL, noise-induced hearing loss; non-NIHL, without NIHL; ROC, receiver operating characteristics; AUC, area under the curve.

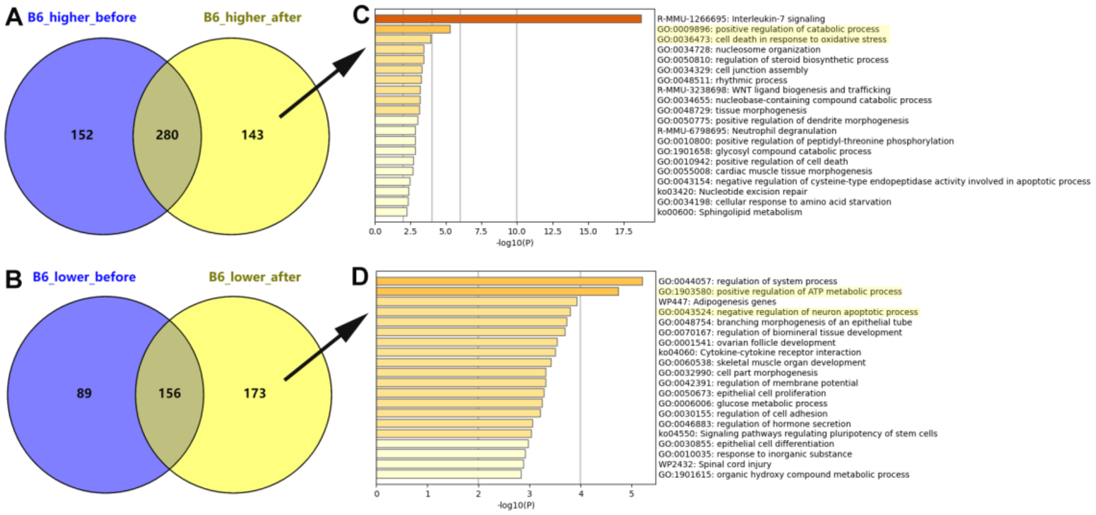


**Figure S4**. The differentially expressed cochleae genes between the susceptible and the resistant mice. (A) The higher expressed cochleae genes in the susceptible B6 mice than the resistant 129X mice before and after noise-exposure. (B) The lower expressed cochleae genes in the susceptible B6 mice than the resistant 129X mice before and after noise-exposure. (C) The pathways associated with the genes which emerged to be higher expressed in susceptible B6 mice than resistant 129X mice after noise exposure. (D) The pathways associated with the genes which emerged to be lower expressed in susceptible B6 mice than resistant 129X mice after noise exposure.
